# IP_3_R at ER-Mitochondrial Contact Sites: Beyond the IP_3_R-GRP75-VDAC1 Ca^2+^ Funnel

**DOI:** 10.1177/25152564231181020

**Published:** 2023-06-22

**Authors:** Peace Atakpa-Adaji, Adelina Ivanova

**Affiliations:** 1 Department of Pharmacology, University of Cambridge, Tennis Court Road, Cambridge, CB2 1PD, UK

**Keywords:** Ca^2+^, endoplasmic reticulum, mitochondria, membrane contact sites, IP_3_ receptor, VDAC1, GRP75

## Abstract

Membrane contact sites (MCS) circumvent the topological constraints of functional coupling between different membrane-bound organelles by providing a means of communication and exchange of materials. One of the most characterised contact sites in the cell is that between the endoplasmic reticulum and the mitochondrial (ERMCS) whose function is to couple cellular Ca^2+^ homeostasis and mitochondrial function. Inositol 1,4,5-trisphosphate receptors (IP_3_Rs) on the ER, glucose-regulated protein 75 (GRP 75) and voltage-dependent anion channel 1 (VDAC1) on the outer mitochondrial membrane are the canonical component of the Ca^2+^ transfer unit at ERMCS. These are often reported to form a Ca^2+^ funnel that fuels the mitochondrial low-affinity Ca^2+^ uptake system. We assess the available evidence on the IP_3_R subtype selectivity at the ERMCS and consider if IP_3_Rs have other roles at the ERMCS beyond providing Ca^2+^. Growing evidence suggests that all three IP_3_R subtypes can localise and regulate Ca^2+^ signalling at ERMCS. Furthermore, IP_3_Rs may be structurally important for assembly of the ERMCS in addition to their role in providing Ca^2+^ at these sites. Evidence that various binding partners regulate the assembly and Ca^2+^ transfer at ERMCS populated by IP_3_R-GRP75-VDAC1, suggesting that cells have evolved mechanisms that stabilise these junctions forming a Ca^2+^ microdomain that is required to fuel mitochondrial Ca^2+^ uptake.

## Highlight

- IP_3_Rs may have a structural role in forming ERMCS
- All IP_3_R subtypes localise to ER–mitochondrial contact sites
- Various tethering complexes and binding partners ensure the stability of ERMCS where IP_3_R delivers Ca^2+^ to the mitochondria

## Introduction

Membrane contact sites (MCS) provide a means of biochemical exchange of signalling mediators between distinct membran e-bound organelles. For cellular homeostasis, intracellular compartments are required to cooperate via functional junctions in order to regulate various processes including Ca^2+^ signalling, lipid synthesis and trafficking (Chang and Liou, 2016, Zaman et al., 2020), glucose homeostasis (Rieusset, 2018), mitochondrial nucleoid transportation (Qin et al., 2020), autophagy (Kohler et al., 2020), organelle dynamics (Prinz, 2014) and various pathological processes (Kim et al., 2022). MCS are characterised as the regions of proximity between two organelles or cellular compartments that do not completely fuse but are held apart at short distances of ∼30 nm by distinct tethering complexes (Fernandez-Busnadiego et al., 2015; Prinz, 2014). First reported over 60 years ago, the endoplasmic reticulum (ER)–mitochondrial junction is one of the most characterised MCS (Copeland and Dalton, 1959). Tethering complexes of various compositions have been identified between the mitochondria and the ER, posing an important need to understand if these signify the occurrence of functionally distinct junctions, or if independent tethering complexes contribute to the maintenance of the same junctions, so facilitating the overall functions of these contact sites.

The composition of the ER–mitochondrial MCS (ERMCS) has been thoroughly reviewed previously (de Brito and Scorrano, 2010, Prinz, 2014, Sassano et al., 2022, Xu et al., 2020). The morphology and composition of these junctions are not static but are dynamic and responsive to changing cellular metabolic demands. Cryogenic-electron microscopy (Cryo-EM) analysis has shown that the length of ERMCS changes in response to nutrient availability (Sood et al., 2014), while biochemical analysis reveals a change in the number of ERMCS during transitions in nutrient availability (Theurey et al., 2016). The onset of ER stress is reportedly marked by an increase in ERMCS and subsequent changes in mitochondrial functions that drive the cellular adaptation to this signal (Bravo et al., 2011). Similarly, a change in ER–mitochondria coupling is also observed following chemically-induced mitochondrial stress (Lopez-Crisosto et al., 2021). Advanced imaging techniques have also shown the dynamism of these junctions, revealing highly motile VAMP-associated protein B (VAPB) positive contact sites that are altered in response to nutrient stress (Obara et al., 2022). In this review, we will discuss the role of ERMCS in shaping the delivery of Ca^2+^ to the mitochondria. Specifically, we will highlight the role and regulation of component proteins that drive the stability and delivery of Ca^2+^ at these junctions.

## ERMCS and Ca^2+^ Signalling

The role of ERMCS in shaping Ca^2+^ signals is one of the most characterised functions of these contact sites. Ca^2+^ must permeate the outer mitochondrial membrane (OMM) and the inner mitochondrial membrane (IMM) to reach the mitochondrial matrix. The OMM has a high Ca^2+^ permeability. This is possibly due to its abundant expression of voltage-dependent anion-selective channel proteins (VDACs) (of which there are 3 subtypes (VDAC1-3)); although VDAC1 is predominantly implicated in mitochondrial Ca^2+^ dynamics, it was shown to selectively co-immunoprecipitate with inositol 1,4,5-trisphosphate receptors (IP_3_R; see later)(De Stefani et al., 2012; Gincel et al., 2001, Rapizzi et al., 2002). There may yet be unidentified Ca^2+^ permeable channels on the OMM that drive its high permeability to Ca^2+^ as the loss of all VDAC isoforms does not affect mitochondrial-dependent cell death, a process thought to occur following mitochondrial Ca^2+^ overload (Baines et al., 2007). Following many years of debate, the mitochondrial Ca^2+^ uniporter (MCU) was identified as the mediator of Ca^2+^ transport through the IMM into the mitochondrial matrix (De Stefani et al., 2011; Kirichok et al., 2004). The Ca^2+^ uptake into mitochondria is now strongly encouraged to involve a tightly regulated multiprotein complex (reviewed in (Giorgi et al. 2018), (De Stefani et al., 2015)). The low affinity of the MCU (K_D_ Ca^2+^ for MCU, ∼20 −30 µM) is evolutionary important to avoid unregulated Ca^2+^ overload of the mitochondria for triggering apoptosis (Bragadin et al., 1979). The accumulation of high concentrations of Ca^2+^ in the mitochondria occurs following the stimulation of Ca^2+^ channels on the ER. This is only made possible due to their close apposition to the ER (Csordas et al., 2010; Rizzuto et al., 1998). Between 5% and 20% of the surface of the mitochondrial network in HeLa cells is in contact with the ER under resting conditions (Rizzuto et al., 1998)*.* The canonical view of the ERMCS protein complex driving Ca^2+^ exchange is the interaction of IP_3_R with VDAC1 via the cytosolic protein linker GRP75. GRP75 is part of the heat shock protein 70 family of proteins and is reported to stabilise the IP_3_R-VDAC1 interaction and therefore promote the formation of ERMCS enabling Ca^2+^ transfer into the mitochondria (Xu et al., 2018). It is important to note that other than at ERMCS, mitochondrial Ca^2+^ uptake dynamics have also been implicated in regulating Ca^2+^ signals at ER–PM membrane contact sites by regulating STIM1 oligomerisation at these junctions (Deak et al., 2014, Nan et al., 2021).

**Figure 1.**
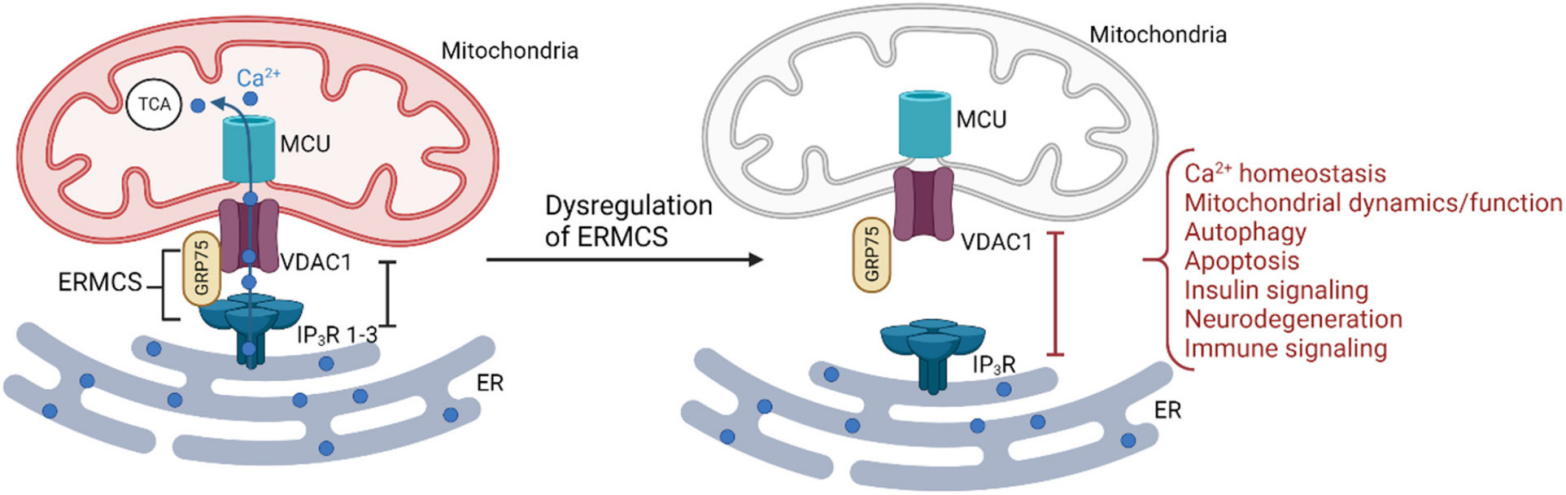
**Ca^2+^ Transfer at ER–Mitochondria Contact Sites Is Important for Cellular Homeostasis.** IP_3_R forms a tightly regulated complex with GRP75 in the cytosol and VDAC1 in the outer mitochondrial membrane to allow preferential access of high Ca^2+^ concentrations to fuel the low-affinity MCU. Ca^2+^ taken up into the mitochondria is important for mitochondrial function and dynamics. Dysregulation of ERMCS abrogates mitochondrial Ca^2+^ uptake with consequences for cellular Ca^2+^ dynamics and various pathophysiological processes.

The transfer of Ca^2+^ at ERMCS not only shapes the spatiotemporal properties of global Ca^2+^ signals but also directly affects mitochondria functions. For example, the activity of three of the important enzymes in the electron transport chain pyruvate dehydrogenase, isocitrate dehydrogenase, oxoglutarate dehydrogenase (OGDH) and α-ketoglutarate dehydrogenase is Ca^2+^-dependent (Denton, 2009). Ca^2+^ signalling at ERMCS is also an important regulator of cellular apoptosis (Kroemer and Reed, 2000). Ca^2+^ overload into the mitochondria is an important trigger for apoptosis leading to mitochondrial swelling, the opening of the mitochondrial permeability transition pore (mPTP) and release into the cytosol of apoptotic factors such as cytochrome c, procaspase 9 and apoptosis-inducing factor 9 (Giorgi et al. 2018, Sukumaran et al., 2021). Furthermore, B cell lymphoma 2 (Bcl-2), mainly resident on the OMM, is an anti-apoptotic protein which reduces the steady-state ER Ca^2+^ concentration and subsequent Ca^2+^ transfer to the mitochondria so enhancing the apoptotic signals (Pinton and Rizzuto, 2006, Sukumaran et al., 2021). Conversely, the pro-apoptotic protein BAX promotes apoptosis by increasing Ca^2+^ transfer to the mitochondria from the ER (Sukumaran et al., 2021). VDAC1 oligomerisation is also implicated in regulating apoptosis (Shoshan-Barmatz et al., 2018). Moreover, a reduction in mitochondrial Ca^2+^ uptake, such as following down-regulation of IP_3_R on the ER, leads to the impairment of cellular bioenergetics and the activation of autophagy, a process where obsolete cellular components are degraded or recycled in lysosomes (Sukumaran et al., 2021). It is therefore not unexpected that disruptions of ERMCS are implicated in the pathophysiology of multiple diseases such as amyotrophic lateral sclerosis (Sakai et al., 2021), Alzheimer's disease (Area-Gomez et al., 2009, Yu et al., 2021), Parkinson's disease (Guardia-Laguarta et al., 2014), Huntington's disease (Kim et al., 2010; Milakovic et al., 2006) and cancer (Simoes et al., 2020) (Figure 1).

## Localisation and Functioning of IP_3_Rs at ERMCS

IP_3_Rs are high-conductance intracellular Ca^2+^ channels, ubiquitously expressed in eukaryotic cells, which open in response to extracellular stimuli. Ca^2+^ signals elicited by IP_3_Rs regulate a vast plethora of biological processes such as motility, neurotransmitter release, apoptosis and gene transcription (Berridge, 2016; Clapham, 2007). IP_3_Rs are predominantly located on the ER but have also been reported to be in the nuclear envelope, plasma membrane, Golgi and secretory vesicles (Dellis et al., 2006; Malviya et al., 1990; Pinton et al., 1998, Yoo and Albanesi, 1990). There are 3 subtypes of IP_3_R, which have a molecular mass of ∼300 kDa and share 60-80% sequence homology, although they are encoded by different genes and expressed differentially in tissues (Foskett et al., 2007; Taylor et al., 1999). The existence of alternative splice variants confers further diversity to IP_3_R expression in cells (Foskett et al., 2007).

Functionally, IP_3_R exists as tetrameric channels which can be homomeric or heteromeric (Alzayady et al., 2013). IP_3_R are co-regulated by binding to IP_3_ and Ca^2+^, with the latter regulating the channel in a biphasic manner such that at low cytosolic concentrations of Ca^2+^ in the presence of IP_3_ activates the channel (Mak and Foskett, 1998) while high Ca^2+^ concentrations conversely inhibit the IP_3_ receptor (Kaftan et al., 1997, Mak and Foskett, 1998). Recent insights into the structure of IP_3_Rs continue to improve our understanding of the structural basis for IP_3_R activation and channel opening (Schmitz et al., 2022). Whilst most cells express more than one IP_3_R subtype, preferential expression of the subtypes has been reported, with IP_3_R1 reported as the predominant subtype in Purkinje cell neurons (Nakanishi et al., 1991), IP_3_R2 in cardiac myocytes (Vervloessem et al., 2015) but also in the liver and epithelium (Klar et al., 2014), and IP_3_R3 in pancreatic β cells (Blondel et al., 1994), testis and endothelial cells (De Smedt et al., 1997). While all three IP_3_R subtypes are regulated by IP_3_ and Ca^2+^, they have different IP_3_ affinities in the order IP_3_R2 > IP_3_R1 > IP_3_R3 (Iwai et al., 2007, Miyakawa et al., 1999; Newton et al., 1994; Tu et al. 2005b, Zhang et al., 2011) and are distinctly modulated by additional signals such as Ca^2+^ and ATP (Mak et al., 2001; Prole and Taylor, 2016, Tu et al. 2005a, Wagner and Yule, 2012; Yoneshima et al., 1997). In systems containing all three IP_3_R subtypes, the composition of IP_3_R channels that participate in Ca^2+^ release remains a question, as minor subtypes could be selectively contributing to the Ca^2+^ signals while the major subtype remains silent (Lock et al., 2018).

The Ca^2+^ signals evoked by IP_3_R occur in a hierarchical manner, with increasing IP_3_ stimulus corresponding to a graded response from local Ca^2+^ events, such as Ca^2+^ puffs involving the opening of a few IP_3_R to global Ca^2+^ waves that spread across the entire cell (Berridge et al., 2000). All IP_3_R subtypes can evoke Ca^2+^ puffs with a similar frequency and amplitude, but IP_3_R2's high affinity for IP_3_ is reflected in the slower kinetics of individual puffs (Lock et al., 2018, Mataragka and Taylor, 2018). The original notion that Ca^2+^ puffs are the building blocks of all modes of Ca^2+^ signalling (Berridge, 1997, Bootman and Berridge, 1996, Bootman et al., 1997, Marchant et al., 1999, Parker et al., 1996) has recently been challenged; it has been suggested that, sustained global signals are evoked by a diffuse mode of Ca^2+^ release distinct from Ca^2+^ puffs which terminate during the early stages of global Ca^2+^ waves (Lock and Parker, 2020). Another layer of complexity in the strict regulation of IP_3_R activity is that not all IP_3_Rs are licensed to release Ca^2+^. The majority of IP_3_Rs in a cell are mobile. However, Ca^2+^ signals originate from a subset of IP_3_R immobilised on actin via KRas-induced actin-binding protein (KRAP). The loss of KRAP reduces the number of immobile IP_3_R clusters in a cell, abrogates Ca^2+^ puffs and global Ca^2+^ signals (Thillaiappan et al., 2021). There are thought to be approximately 8 IP_3_Rs in a licensed IP_3_R cluster from which elementary Ca^2+^ events occur (Thillaiappan et al., 2017, 2021). However, further analysis has suggested that KRAP may license individual IP_3_R channels rather than the entire cluster as a signalling unit (Vorontsova et al., 2022). Furthermore, cyclic AMP response element-binding protein (CREB) may affect IP_3_R1 licensing by regulating the expression of KRAP in HEK293 cells (Arige et al., 2021). Even with our increased knowledge of the structure and function of IP_3_R, there continues to be active research on the fine-tuning of its licensing, activity and spatial localisation.

### All IP_3_R Subtypes Localise to ER–Mitochondrial Contact Sites

With different biophysical properties of the IP_3_R subtypes, there has been a long interest in the subtype-specific regulation of ER–mitochondrial contact sites. Initial analysis into the importance of subtype selectively in the role of IP_3_R at ER–mitochondria MCS in CHO-KI and HEK293 cells has suggested a preferentiality for IP_3_R3 in delivering Ca^2+^ to mitochondria and subsequent regulation of apoptosis (Mendes et al., 2005). Furthermore, Mendes et al. using confocal microscopy suggested that IP_3_R3 colocalises more extensively with mitochondria compared to the other 2 subtypes (Mendes et al., 2005). For many years it was thus considered that IP_3_R3 selectively interacts with mitochondria at ERMCS and is the required mediator of apoptotic Ca^2+^ overload. Nonetheless, there have been isolated studies showing the roles of the other IP_3_R subtypes at ERMCS in different biological settings. Recombinant expression of IP_3_R1 was shown to enhance Ca^2+^ accumulation in mitochondria in the rat liver and HeLa cells (Szabadkai et al., 2006). Furthermore, IP_3_R1 forms a tripartite complex with GRP75 and VDAC1 modulating diabetic atrial remodelling (Yuan et al., 2022). In astrocytes, IP_3_R2 was found to be accumulated together with mitochondria at the sites of regenerative Ca^2+^ wave initiation (Simpson et al., 1998). However, in a systematic study, Bartok et al. showed that all IP_3_R subtypes are competent to restore contacts between the ER and mitochondria in a null IP_3_R background with compromised ERMCS (Bartok et al., 2019). Super-resolution analysis shows that all IP_3_R subtypes can form clusters near to the mitochondria, but IP_3_R2 may be more enriched at ER–mitochondrial junctions in DT40 cells. Whilst all IP_3_R subtypes were found to be competent in transferring Ca^2+^ to mitochondria, IP_3_R2 displayed higher efficacy in mediating Ca^2+^ transfer from the ER to the mitochondria in DT40 cells (Bartok et al., 2019). This work further suggests that IP_3_R1-mediated Ca^2+^ transfer to the mitochondria may be predominantly sustained by Ca^2+^ entry across the plasma membrane in DT40 cells (Bartok et al., 2019). Therefore, there is growing evidence of the localisation of all IP_3_R subtypes at ERMCS (Table 1). Further assessment of the context-specific contribution of IP_3_R subtypes to ERMCS formation and function is required to address the potential involvement of all three subtypes in junction stability and Ca^2+^ transfer from the ER to the mitochondria.

**Table 1.** Summary of IP_3_R subtype identification at ERMCS in different cell types.

Subtype	Cell line	Comment	Reference
IP_3_R1	DT40	IP_3_R1 may increase [Ca^2+^]_mito_, fuelled by Ca^2+^ influx from PM	(Bartok et al., 2019)
	Rat liver	IP_3_R1 forms a complex with GRP75 and VDAC1	(Szabadkai et al., 2006)
	HeLa	IP_3_R1 forms a complex with GRP75 and VDAC1	(Szabadkai et al., 2006)
IP_3_R2	Astrocytes	IP_3_R2 sits with mitochondria at the sites of regenerative Ca^2+^ signals	(Simpson et al., 1998)
	DT40	Suggests all IP_3_R can exist at ERMCS but IP_3_R2 most effective in Ca^2+^ transfer to mitochondria	(Bartok et al., 2019)
IP_3_R3	HeLa	IP_3_R3 forms a complex with GRP75 and VDAC1	(Gomez-Suaga et al., 2017)
	HEK	siRNA to IP_3_R3 selectively reduces [Ca^2+^]_mito_	(Mendes et al., 2005)
	CHO	siRNA to IP_3_R3 selectively reduces [Ca^2+^]_mito_	(Mendes et al., 2005)
	C57B6/J mice brain slices	IP_3_R3 at MAM fractions with VDAC1, Tom70 and VAPB	(Filadi et al., 2018)

### IP_3_Rs Have a Structural Role in Forming ERMCS

Recent evidence suggests that IP_3_Rs may play a structural role in ERMCS formation which is independent of their ability to deliver Ca^2+^ from the ER for mitochondrial uptake via the MCU. HEK293 cells are reported to show limited ER–mitochondrial Ca^2+^ transfer, but tight IP_3_R-dependent ERMCS (Katona et al., 2022). HEK293 cells lacking IP_3_R showed fewer regions of close apposition (<20 nm) between the ER and mitochondria and re-expression of IP_3_Rs including a non-Ca^2+^ conducting mutant in HEK293 cells rescued the contacts between the ER and mitochondria (Katona et al., 2022). Recruitment of mobile IP_3_R at ER–mitochondrial contacts has been shown to license the IP_3_Rs to rapidly deliver Ca^2+^ to the mitochondria and increase the activity of mitochondrial Ca^2+^-sensitive dehydrogenases (Katona et al., 2022). Analysis using drug-inducible ER–mitochondrial linkers showed that ERMCS junctions formed more quickly in WT DT40 cells compared to IP_3_R knock-out cells where IP_3_R-mediated Ca^2+^ release was completely attenuated (Bartok et al., 2019). This further suggests that IP_3_R are not situated at junctions with mitochondria solely for the provision of Ca^2+^, but are required for junction assembly. IP_3_R-mediated regulation of the gap length at specialised junctions is independent of their Ca^2+^ flux property as pore-dead IP_3_Rs are equally as competent in restoring tight junctions. Close ER–mitochondria proximity nonetheless remains key for Ca^2+^ transfer to the mitochondria as forcing the mitochondria to the PM and away from the ER disrupts IP_3_R-mediated Ca^2+^ transfer into the mitochondria. Such proximity is likely maintained by several tethering complexes acting synergistically.

### Co-Regulators of the IP_3_R-GRP75-VDAC1 complex at ERMCS

**Figure 2.**
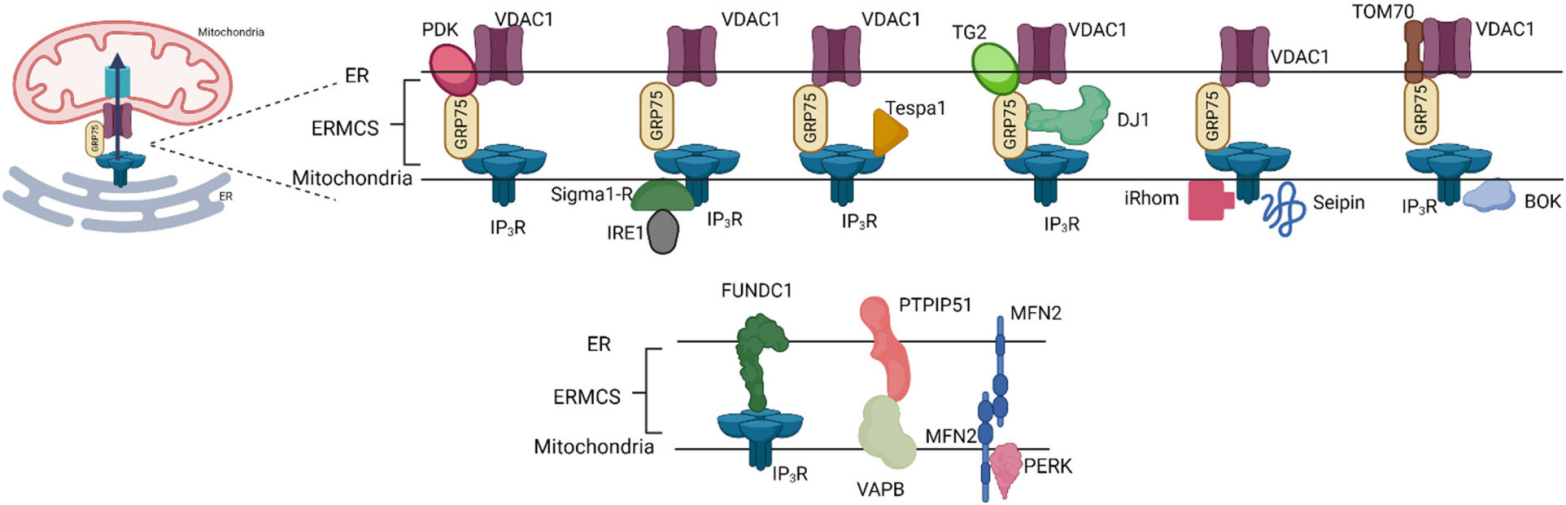
**Composition of ER–Mitochondrial Contact Sites Where Ca^2+^ Exchange Occurs.** Various binding partners and regulators of the IP_3_R-GRP75-VDAC1 complex have been described. These act either by binding directly to one or more of the components (top) or by stabilising the ERMCS (bottom) thereby allowing IP_3_R to remain localised preferentially to provide Ca^2+^ for the MCU.

Although the IP_3_R–VDAC–GRP75 interaction in the canonical tethering complex is often reported at the ERMCS Ca^2+^ microdomain, these junctions can be modulated by other membrane-anchored or cytosolic binding partners (Figure 2).

#### Tespa1

Thymocyte-expressed positive selection-associated gene 1 (*Tespa1*) is an important regulator of T cell development in the thymus which shares sequence homology (including the double phenylalanine motif that interacts with IP_3_Rs) with KRAP (Matsuzaki et al., 2012, Thillaiappan et al., 2021). Although it lacks the actin-binding domain of KRAP, Tespa1 has been shown to interact with IP_3_Rs as well as GRP75 in T and B lymphocytes (Matsuzaki et al., 2012, Matsuzaki et al., 2013). The loss of Tespa1 leads to a reduction in both cytosolic and mitochondrial Ca^2+^ signals following T-cell receptor stimulation (Matsuzaki et al., 2013). This would suggest that Tespa1 functions in these cells to regulate IP_3_R activity at ERMCS (Matsuzaki et al., 2012).

#### Sigma-1 Receptor

The ER chaperone protein sigma-1 receptor is an important regulator of Ca^2+^ signalling at ERMCS. The sigma-1 receptor is thought to remain bound to another chaperone protein binding immunoglobulin protein (BiP) under physiological conditions. However, upon ER stress, such as during ER Ca^2+^ depletion, sigma-1 dissociates from BiP and translocates to ERMCS, stabilising IP_3_R3 at these junctions and promoting the prolonged release of Ca^2+^ from the ER into the mitochondria (Hayashi and Su, 2007). Other functions of the sigma-1 receptor at ERMCS include stabilising the ER stress sensor inositol-requiring enzyme 1 (IRE-1) and promoting its ability to respond to mitochondria-derived reactive oxygen species during ER stress (Mori et al., 2013). Furthermore, the sigma-1 receptor may regulate dendritic spine formation by modulating the free radical formation around ERMCS to dampen caspase-3 activation and consequent inactivation of Rac GTPase via the degradation of guanine nucleotide exchange factor (Tsai et al., 2009). Sigma-1 receptors functioning at ERMCS are implicated in the pathophysiology of multiple neurodegenerative diseases (Nguyen et al., 2017).

#### BOK

B cell lymphoma 2 (BCL-2) ovarian killer (BOK) is an ER-resident pro-apoptotic protein. It has been shown to bind IP_3_Rs, regulating both Ca^2+^ dynamics at ERMCS under physiological conditions, and following a stimulus (Carpio et al., 2021). BOK is also shown to regulate the stability and composition of proteins at ERMCS (Carpio et al., 2021). The loss of BOK leads to a decreased localisation of IP_3_R1, IP_3_R3 and the sigma-1 receptor at ERMCS as assessed by microscopy (Carpio et al., 2021). Furthermore, whilst the amount of VDAC1 is unchanged following the loss of BOK in pure mitochondria-associated membrane (MAM) fractions, the amount of IP_3_R1 and IP_3_R3 is significantly attenuated (Carpio et al., 2021). Conversely, overexpression of MCL-1 and BOK transmembrane domains in HeLa cells increases ERMCS populated by IP_3_R and VDAC as assessed via proximity ligation assay (Lucendo et al., 2020). The BOK-IP_3_R interaction at ERMCS is implicated in the regulation of apoptosis in these cells (Carpio et al., 2021). Altogether, these suggest BOK plays a role in the recruitment and localisation of IP_3_R at ERMCS.

#### Tom70

The translocase of the outer membrane (TOM) family of proteins accounts for a significant proportion of the OMM protein content. TOM proteins, together with the translocase of the inner membrane (TIM), are key regulators of the trafficking of mitochondrial proteins encoded in the nucleus (Chacinska et al., 2009). Unlike TOM20 which has a uniform distribution across the OMM, TOM70 appears more punctate, coinciding with ERMCS contact sites identified with an engineered split-GFP construct (Filadi et al., 2018). TOM70 was also found to be present in pure MAM fractions physically bind to IP_3_R2 (as assessed by co-immunoprecipitation analysis) and regulate IP_3_R-mediated Ca^2+^ transfer and cellular bioenergetics (Filadi et al., 2018). Of note, the depletion of TOM70 did not affect the number of ERMCS junctions detected (Filadi et al., 2018). This suggests that TOM70 is not structurally required in the assembly of the junction but may be involved in ‘trapping’ IP_3_R at these junctions.

#### Transglutaminase Type 2 (TG2)

Transglutaminase type 2 (TG2) is an important mediator of post-translational modifications of proteins and localises to various cellular compartments including the cytosol, mitochondria and nucleus (Szondy et al., 2017). Mass spectrometry reveals that TG2 interacts with multiple proteins on the ER and mitochondria including BiP, TOM70 and GRP75 (D’Eletto et al., 2018). TG2 was found to immunoprecipitate with GRP75 and was also enriched in pure MAM fractions (D’Eletto et al., 2018). Overexpression of TG2 reduces the IP_3_R3–GRP75 interaction in MEFs, suggesting that TG2 is a negative regulator of IP_3_R-GRP75 (D’Eletto et al., 2018). TG2 also modulates the composition of ERMCS and mitochondrial Ca^2+^ uptake following agonist stimulation (D’Eletto et al., 2018). Although the IP_3_R3–GRP75 association is increased in the absence of TG2, the overall ERMCS number is reduced (D’Eletto et al., 2018). One argument is that the increase in the IP_3_R3–GRP75 interaction occurs as a compensatory mechanism for the reduction in physical coupling between the ER and mitochondria. It remains to be elucidated if TG2 is a physical tether or only a regulatory partner at these ERMCS.

#### iRhom

iRhom, a catalytically-dead member of the rhomboid family of serine proteases is best characterised as a cofactor of ADAM metallopeptidase domain 17 (ADAM17) (Dulloo et al., 2019). Although not exclusively, iRhoms which has 2 subtypes (iRhom1/2) can be found on the ER and have been implicated in regulating the transfer of Ca^2+^ to the mitochondria via IP_3_Rs in ER stress (Dulloo et al., 2022). Depletion of iRhom1/2 under ER stress leads to defective mitochondrial membrane potential and abrogated IP_3_R-mediated cytosolic and mitochondrial Ca^2+^ signals (Dulloo et al., 2022). Although iRhoms were shown to interact with IP_3_Rs, it is not clear if they reside within ERMCS, or if they indirectly affect mitochondrial Ca^2+^ uptake by globally attenuating IP_3_R activity under ER stress.

#### Seipin

Seipin is an ER resident protein implicated in lipodystrophy (Combot et al., 2022). The observation that patient-derived lymphoblast showed perturbed mitochondrial morphology prompted further investigation into the possible roles of this protein at ERMCS (Combot et al., 2022). Seipin is reported to be enriched at ERMCS populated by IP_3_R, VDAC and the sarco/endoplasmic reticulum Ca^2+^-ATPase (SERCA) during starvation (Combot et al., 2022). Seipin deficiency leads to attenuation of the uptake of mitochondrial Ca^2+^ following IP_3_R stimulation and impaired mitochondrial metabolism and dynamics in A431 cells (Combot et al., 2022).

#### Pyruvate Dehydrogenase Kinases (PDKs)

Pyruvate dehydrogenase kinases (PDKs) are serine/threonine kinases mainly located in the mitochondria where they regulate the activity of pyruvate dehydrogenase (PDH) in glycolysis (Wang et al., 2021). There are four isoforms of PDK (PDK 1-4), showing broad tissue expression. Using proximity ligation assays and cellular fractionation analysis, PDK4 alone was shown to localise and interact with IP_3_R1–VDAC–GRP75 at ERMCS in skeletal muscle cells (Thoudam et al., 2018). Overexpression of PDK4 enhances the IP_3_R1–VDAC1–GRP75 interaction at ERMCS (Thoudam et al., 2018). The kinase activity of PDK4 is required for this enhancement as the pharmacological inhibition of its kinase activity, or the use of a kinase-dead mutant, does not show this enhancement (Thoudam et al., 2018). Inhibition of PDK4 in these cells also attenuates IP_3_R-mediated Ca^2+^ transfer to the mitochondria without affecting the ER store content (Thoudam et al., 2018). Using mice models, PDK4 has been shown to be increased in obesity, leading to the regulation of ERMCS formation and Ca^2+^ transfer to the mitochondria (Thoudam et al., 2018). This suggests PDK4 as a structural tether and stabiliser of IP_3_R-positive ERMCS in these specialised cells. PDKs are traditionally known to reside in the mitochondrial matrix (Wang et al., 2021), therefore the mechanism by which they interact and couple with IP_3_R1, GRP75 and VDAC in MAM fractions remains unclear. Furthermore, PDK4 can be found in other cell types such as the liver, kidneys and pancreatic cells (Moon et al., 2012), but it remains to be determined if it also interacts at ERMCS in these other cells.

#### Other Co-Regulators of ERMCS Where Ca^2+^ Exchange Occurs

Other regulators of ERMCS important for Ca^2+^ exchange have been described in metabolic regulation, neurodegenerative disease models and in cancer. In metabolic regulation, abrogation of the tethers VAPB on the ER membrane and protein tyrosine phosphatase interacting protein 51 (PTPIP51) on the mitochondrial membrane at ERMCS that regulate autophagy attenuates the IP_3_R3–VDAC1 interaction and subsequent IP_3_R mediated Ca^2+^ signals to the mitochondria (Gomez-Suaga et al., 2017). Furthermore, the deletion of PTP1P51 coiled-coil domain which affects its localisation at ERMCS, abrogates IP_3_R-mediated Ca^2+^ delivery to the mitochondria (Mórotz et al., 2022). In cardiomyocytes, the mitochondrial resident protein FUN14 domain containing 1 (FUNDC1) binds to IP_3_R2 to regulate ERMCS and modulate Ca^2+^ exchange at these junctions (Wu et al., 2017).

In neurodegenerative disease models, the loss of function mutation in Niemann-Pick type C1 (NPC1) changes the spatial distribution of IP_3_R1, potentiates GPCR-mediated Ca^2+^ signals, and promotes IP_3_R-mediated mitochondrial Ca^2+^ uptake causing cytotoxicity (Tiscione et al., 2021). Furthermore, DJ-1 encoded for by PARK7 is a cytosolic and nuclear protein implicated in early onset Parkinson's disease. PARK7 which translocates to ERMCS to interact with the IP_3_R–GRP75–VDAC1 complex to regulate both the integrity of the ERMCS and Ca^2+^ transfer at these junctions (Liu et al., 2019). DJ1 regulates the spatial localisation of IP_3_R, with the loss of DJ1, leading to the aggregation of IP_3_R via an unknown mechanism (Liu et al., 2019). Actin polymerisation via the ER-anchored inverted formin 2 (INF2) results in an increase in mitochondrial Ca^2+^ and further change in the mitochondrial morphology via dynamin-related protein 1 (Drp1) recruitment (Chakrabarti et al., 2018).

In adrenocortical carcinoma, fetal and adult testis-expressed 1 (FATE1), which encodes a cancer-testis antigen has been shown to localise to MAMs (Doghman-Bouguerra et al., 2016). There it decreases ERMCS and negatively modulates the transfer of IP_3_R-mediated Ca^2+^ to the mitochondria. In addition, Furthermore, FATE1 has also been suggested to attenuate apoptosis in these adrenocortical carcinoma cells (Doghman-Bouguerra et al., 2016). Upregulation of FATE1 in cancers may occur to drive uncoupling of the ER and mitochondria, attenuate Ca^2+^ delivery and increase the resistance to cell death in these cancer cells.

## Perspectives and Concluding Remarks

As highlighted, multiple regulators of the IP_3_R-GRP75-VDAC complex at ERMCS mediate Ca^2+^ exchange in various cell types. This poses the question of how ERMCS are regulated? Furthermore, the dogma that the IP_3_R–GRP75–VDAC tripartite complex forms a funnel through which Ca^2+^ is exchanged from the ER to the mitochondria, requires closer scrutiny. Firstly, there is evidence that other binding partners can interact with IP_3_R to form ERMCS where Ca^2+^ exchange occurs, as is the case for IP_3_R interacting with the AKAP1 transmembrane domain or TOM70 on the mitochondrial membrane (Katona et al., 2022). Although it is important to note that TOM70 has been shown to interact directly with the IP_3_R–GRP75–VDAC complex (SECTION 3.3); therefore, TOM70 may well be a coincident reporter of the same IP_3_R–GRP75–VDAC junctions. Secondly, disruption of other tethering complexes such as between VAPB and PTPIP51 on the OMM, also disrupts Ca^2+^ uptake into the mitochondria (De Vos et al., 2012). This suggests a model where tethering complexes work synergistically to promote ERMCS where Ca^2+^ exchange can occur. Indeed, overexpression of VAPB and PTP1P51 was shown to increase the IP_3_R3–VDAC1 interaction in HeLa cells and Ca^2+^ exchange at these junctions (Gomez-Suaga et al., 2017). Furthermore, the knockdown of mitofusin-2 which tethers at ERMCS via interaction with either mitofusin 1 or 2 on the OMM also regulates the stability of the junctions, with its ablation leading to attenuated mitochondrial Ca^2+^ uptake (de Brito and Scorrano, 2008, Naon et al., 2016). This is not without controversy, since MFN2 has been postulated to act in the opposite direction where it reduces the stability of ERMCS and its reduction increases Ca^2+^ transfer to the mitochondria (Filadi et al., 2015). Thirdly, local Ca^2+^ transfer at ERMCS can be circumvented using a sufficiently high concentration of a rapid Ca^2+^ chelating agent such as ethylene glycol-bis(β-aminoethyl ether)-N,N,N′,N′-tetraacetic acid (EGTA) (Csordas et al., 1999). A tight Ca^2+^ microdomain synonymous with a funnel may be impenetrable by buffering agents as Ca^2+^ can be channelled through to the mitochondria regardless of the buffering activity in the cytosol.

We suggest that the ER–mitochondrial Ca^2+^ microdomain is maintained by a complex of different tethers and binding proteins working synergistically to promote ERMCS contact sites where IP_3_R sits to power the low-affinity mitochondrial Ca^2+^ uptake mechanism. Further work is required to elucidate the interdependence of various tethering complexes on mitochondrial Ca^2+^ uptake and their role in health and disease. Whilst current technologies are limited in their capabilities, it is also important to assess how the individual tethering complexes at ERMCS change simultaneously in response to metabolic demands, as it will help answer the longstanding questions around the role of the multiple complexes found at these junctions.

## Abbreviations

BAX: Bcl-2-associated X protein
Bcl-2: B cell lymphoma 2
BOK: Bcl-2 related ovarian killer
Ca^2+^: calcium
CHO: Chinese hamster ovary cell
CREB: cyclic AMP-response element-binding protein
Cryo-EM: cryogenic electron microscopy
EGTA: tetra(acetoxymethyl Ester)
ER: endoplasmic reticulum
ERMCS: ER-mitochondrial membrane contact sites
FUNDC1: FUN14 domain containing 1
green: fluorescent protein
GPCR: G protein-coupled receptor
GRP 75: glucose-regulated protein 75
HEK: human embryonic kidney
IMM: inner mitochondrial membrane
INF2: inverted formin 2
IP_3_R: inositol 1,4,5-trisphosphate
KRAP: KRas-induced actin binding protein
MAM: mitochondria-associated membrane
MCS: membrane contact sites
MCU: mitochondrial Ca^2+^ uniporter
mPTP: mitochondrial permeability transition pore
NPC1: Niemann-Pick type C1
OGDH: oxoglutarate dehydrogenase
OMM: outer mitochondrial membrane
PDK: pyruvate dehydrogenase kinases
PM: plasma membrane
PTPIP51: protein tyrosine phosphatase interacting protein 51
SERCA: sarco/endoplasmic reticulum Ca^2+^-ATPase
STIM1: stromal interaction molecule 1
Tespa1: thymocyte-expressed positive selection-associated gene 1
TG2: transglutaminase type 2
TIM: translocase of the inner membrane
TOM70: translocase of the outer membrane 70
VAPB: VAMP associated protein B
VDAC: voltage-dependent anion-selective channel proteins
WT: wild type
